# Prediction of human missense variant effects from functional evidence

**DOI:** 10.21203/rs.3.rs-7536763/v1

**Published:** 2025-10-03

**Authors:** Barış Kayaalp, Kerem Çil, Clément Conil, Aurélie Cobat, Meltem Ece Kars, Yuval Itan, Jean-Laurent Casanova, Tayfun Özçelik

**Affiliations:** 1 Department of Molecular Biology and Genetics, Bilkent University, 06800 Ankara, Türkiye; 2 Laboratory of Human Genetics of Infectious Diseases, Necker Branch INSERM U1163, Necker Hospital for Sick Children, 75015 Paris, France; 3 Imagine Institute, University of Paris, 75015 Paris, France; 4 St Giles Laboratory of Human Genetics of Infectious Diseases, Rockefeller Branch, Rockefeller University, New York, NY 10065, USA; 5 Charles Bronfman Institute of Personalized Medicine, Icahn School of Medicine at Mount Sinai, New York, NY 10029, USA; 6 Department of Genetics and Genomic Sciences, Icahn School of Medicine at Mount Sinai, New York, NY 10029, USA; 7 Mindich Child Health and Development Institute, Icahn School of Medicine at Mount Sinai, New York, NY 10029, USA; 8 The Windreich Department of Artificial Intelligence and Human Health, Icahn School of Medicine, Mount Sinai, New York, NY 10029, USA; 9 Pediatric Immunology-Hematology Unit, Necker Hospital for Sick Children, 75015 Paris, France; 10 HHMI, Rockefeller University, New York, NY 10065, USA

**Keywords:** Variant Effect Predictor, Missense Variants, Inborn Errors of Immunity, PheWAS, Genetic Epidemiology

## Abstract

Prediction of missense variant effects remains the critical bottleneck in disease gene identification and clinical interpretation. Current predictors rely on clinical outcomes or population patterns, rather than direct measures of functional impact, leading to limited generalizability and data circularity. We present FuncVEP, the first family of variant effect predictors trained exclusively on balanced and diverse functional data, providing a direct representation of functional effect. FuncVEP generalizes across contexts, outperforming 47 existing predictors on both clinical and functional benchmarks, improving the accuracy from 82% to 93% and reducing uncertain classifications from 11% to 2%. To illustrate its utility in gene discovery, we applied FuncVEP to 490 inborn errors of immunity genes in the UK Biobank and Mount Sinai Million Health Discoveries Program, identifying 50 novel gene–phenotype associations. FuncVEP provides a robust, scalable solution for variant interpretation, advancing both diagnostic precision and gene discovery.

Rapid and large-scale detection of human genetic variation has transformed medical research and diagnostics^1,2^. However, interpretation has lagged behind discovery, particularly for missense variants, which account for nearly half of protein-coding diversity^3,4^. Most missense variants remain classified as variants of uncertain significance (VUS), and they account for approximately 84% of the most ambiguous subclass, VUS-mid^5^. This pervasive uncertainty limits the utility of sequencing data for both clinical diagnostics and population-scale studies, underscoring the urgent need for predictive frameworks capable of resolving missense interpretation with high accuracy. Addressing this gap is essential for translating genomic discovery into clinical and research progress.

Missense variant classification relies on three sources of evidence: clinical databases^6^, functional assays^7^, and variant effect predictors (VEPs)^8^; but each has significant limitations. Clinical databases and functional assays currently cover only a small fraction—approximately 0.2% and 0.1%, respectively—of the ~75 million possible missense variants in humans. While functional assays are reliable, their application remains constrained by scalability, cost, and labor intensity, which at present prevents them from closing the interpretive gap at genome scale. VEPs aim to bridge this gap. They are classified into three categories—clinical-trained, population-tuned, and population-free predictors—that reflect different levels of data circularity risk^9^. Clinical-trained predictors (e.g., ClinPred, REVEL) learn directly or indirectly from human variants labeled pathogenic/benign from databases such as ClinVar. These methods can perform well on clinical benchmarks but are most at risk of circularity when evaluated on or combined with clinical or population evidence. Population-tuned predictors avoid direct clinical labels but incorporate human allele-frequency information during tuning, optimization, or scaling. This group carries a lower but still meaningful risk of circularity, particularly in the classification of benign variants. AlphaMissense, for instance, is a semi-supervised protein language model that uses human allele frequencies as weak labels during calibration, which limits the detection of common yet damaging variants. Population-free predictors (e.g., ESM1b, SIFT) do not use human population data and are, in principle, free of circularity when assessed on clinical or population-derived variants. However, they generally underperform compared to population-tuned and clinical-trained models, especially when evaluated on clinical datasets.

VEPs are prone to double counting when they incorporate evidence used in the American College of Medical Genetics and Genomics (ACMG) framework, such as allele frequency or splicing scores, artificially boosting classification strength and reducing generalizability^10^. Moreover, predictors trained on clinical endpoints often show reduced concordance on functional datasets such as multiplexed assays of variant effect (MAVEs), highlighting a gap between pathogenicity labels and biochemical function^9,11^. This reflects a broader conceptual issue in VEP development, which is the distinction between a variant’s functional effect on the encoded protein and its clinical pathogenicity. Functional effect refers to the molecular consequence— such as loss-of-function (LOF), gain-of-function (GOF), hypomorph, or neomorph—as measured by experimental assays. Clinical pathogenicity is an organism-level statement that a variant causes disease for a specific phenotype. Translation of functional alterations to organism-level pathogenicity is context dependent^12^, therefore, using clinical annotations as training labels for functional-effect prediction is conceptually flawed.

The primary bottlenecks in missense variant prediction are therefore circularity, limited generalizability across datasets, and indirect modeling of functional impact. We hypothesize that a predictor trained exclusively on diverse and balanced functional data can overcome these limitations while providing accurate, genome-wide estimates of variant effect. Yet, to date, no existing tool has been trained solely on functional evidence.

To test this hypothesis, we developed the Functional-trained Variant Effect Predictor (FuncVEP), a family of VEPs trained on high-quality functional data and explicitly designed to exclude evidence used elsewhere in ACMG frameworks. We show that this design eliminates the core challenges outlined above and enables superior generalization across functional, clinical, *de novo*, and somatic driver mutation datasets.

To assess the translational utility of this approach, we applied FuncVEP to 490 inborn error of immunity (IEI) genes in the UK Biobank. By coupling FuncVEP’s functional predictions with phenome-wide association studies (PheWAS), we identified novel genotype–phenotype associations and assembled a disease-associated missense resource, illustrating how functional effect prediction, when scaled to population biobanks, can accelerate discovery and support precision medicine. An interactive web portal of FuncVEP is available at https://funcvep.bilkent.edu.tr.

## Results

### Predicting the functional effects of all possible missense variants in humans

**Fig. 1 |:**
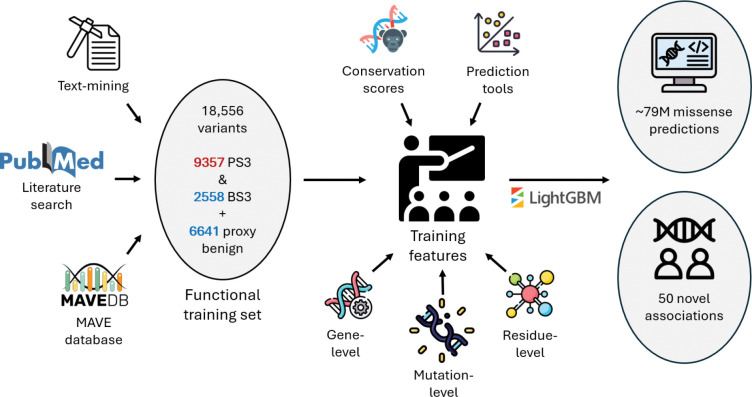
FuncVEP workflow for training, variant effect prediction and gene discovery. High-confidence functional data were aggregated from three sources: text-mined evidence, manually curated literature, and MAVEs. These were combined with proxy benign variants from gnomAD to construct a balanced and diverse functional training dataset (18,556 missense variants: 9,357 PS3-damaging, 2,558 BS3-neutral, and 6,641 proxy benign). FuncVEP models were trained using a wide range of features, including gene-, mutation- and residue-level metrics, conservation scores, and outputs from existing VEPs. The trained models were used to predict the functional impact of all possible missense variants in humans and to enable downstream analyses, including the discovery of novel gene–phenotype associations.

To predict the functional effects of missense variants as damaging or neutral, we constructed a balanced and diverse functional dataset of 18,556 missense variants from 1,576 genes aggregated from high-confidence sources: MAVEs, literature-curated annotations, and text-mined functional evidence (Supplementary Table 1; Supplementary Information: Functional Dataset). We trained LightGBM classifiers using up to 571 features: 45 VEP scores, 13 conservation metrics, 17 structural features, 7 residue constraint scores, 20 amino acid substitution matrices, 4 protein language model–derived features, and 465 gene-level annotations such as constraint, knockout, and expression data^13^ (Supplementary Table 2). To prevent circularity stemming from VEP scores, we omitted the corresponding scores for variants directly or indirectly used in the training of incorporated predictors and applied per-gene label balancing. We trained three models with varying features: (1) CTI (clinical-trained tools included) incorporated all available features, (2) CTE (clinical-trained tools excluded) precluded clinical-trained predictors, and (3) SP (single predictor) excluded all VEP-derived features entirely. These models were then used to generate functional effect predictions as damaging or neutral for all possible ~75 million missense variants in humans (Figure 1). This resulted in the prediction of approximately 26 million damaging and 53 million neutral variants. To directly compare functional and clinical training strategies, we developed mirror models called ClinVEP, trained on a balanced clinical dataset of 11,629 variants from 1,607 genes, using the same feature sets as their corresponding FuncVEP models. The clinical dataset comprised high-confidence (≥2-star) ClinVar^6^ variants with conclusive classifications (Supplementary Table 1). Importantly, the FuncVEP and ClinVEP frameworks were designed to exclude their own training variants from prediction, ensuring that no scores are generated for these variants and eliminating variant-level circularity in all benchmarks.

### Feature importance analysis reveals predictive power of protein language models

**Extended Data Fig. 1.**
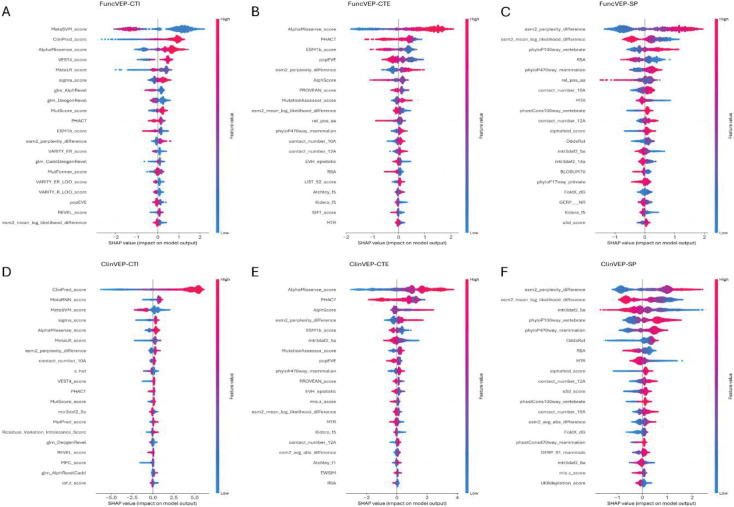
SHAP summary plots of feature impact on model output. SHAP values quantify the contribution of each feature to individual predictions. For each model, the top 20 features ranked by overall impact are shown on the y-axis, and the x-axis represents the magnitude and direction of impact. Each point corresponds to one variant, with feature values encoded by color (red = high feature value, blue = low feature value). Points to the right indicate contributions toward predicting a variant as damaging; points to the left indicate contributions toward predicting a variant as neutral. For example, high values of protein language model–derived features (e.g., ESM2 perplexity difference) tend to shift predictions toward damaging. (A) FuncVEP-CTI, (B) FuncVEP-CTE, (C) FuncVEP-SP, (D) ClinVEP-CTI, (E) ClinVEP-CTE, and (F) ClinVEP-SP.

**Fig. 2 |:**
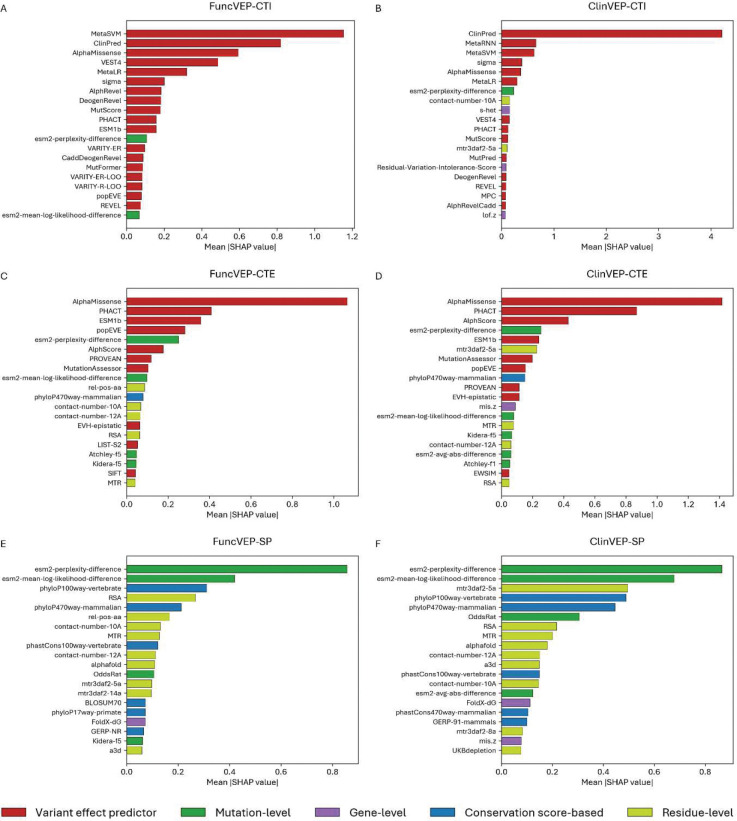
Importance of the top 20 features used by FuncVEP and ClinVEP. Bars represent the top 20 features for each model, ranked by mean absolute SHAP value (features with the greatest contribution appear at the top). The x-axis reflects the average magnitude of each feature’s impact on predictions. Panels on the left display models trained on functional data, while panels on the right show models trained on clinical data: (A) FuncVEP-CTI, (B) ClinVEP-CTI, (C) FuncVEP-CTE, (D) ClinVEP-CTE, (E) FuncVEP-SP, (F) ClinVEP-SP. Differences in feature ranking and type illustrate how each model leverages different biological signals and predictor scores under distinct feature-set constraints.

Feature importance analysis evaluates how much each input feature contributes to a model’s predictions, revealing which biological signals or prediction tools drive its performance. Using the SHAP analysis, we observed that, in CTI models, clinical-trained VEP scores dominated, and two of the four protein language model-derived features, perplexity and mean log-likelihood difference, ranked among the top 20^14^ (Figure 2A, B). In CTE models, AlphaMissense and PHACT ranked highest while structural features showed increased contribution. All four protein language model features ranked among the top 20, reflecting the higher reliance of the model on primary biological context (Figure 2C, D). In SP models, protein language model-derived followed by structural and conservation-based features, dominated the top positions (Figure 2E, F). Protein language model-derived features displayed 85% and 560% increased importance in CTE and SP respectively, compared to CTI (Extended Data Fig. 1 & Supplementary Table 3). This indicates that, when clinical-trained predictors are excluded, the models increasingly leverage intrinsic biological signals, particularly those learned by protein language models, to achieve high performance.

### FuncVEP outperforms existing VEPs on both functional and clinical data

**Extended Data Fig. 2.**
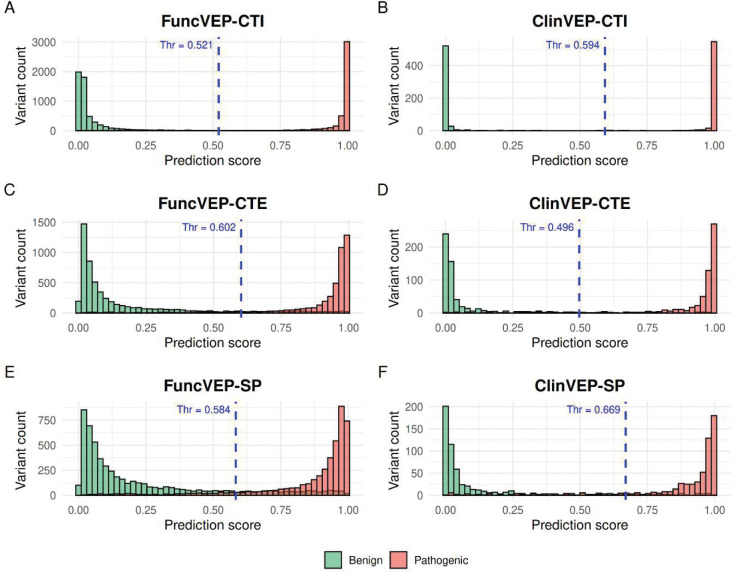
Distribution of model scores and decision thresholds on the clinical dataset. Histograms display the distribution of pathogenic (red) and benign (green) variants from the clinical dataset across the output scores of each model, illustrating the degree of separation between the two classes. The x-axis shows the model output score, and the y-axis indicates the number of variants in each score bin. Blue dashed lines represent the optimized binary decision thresholds used for classification, with the corresponding threshold values labeled above each line. (A) FuncVEP-CTI, (B) ClinVEP-CTI, (C) FuncVEP-CTE, (D) ClinVEP-CTE, (E) FuncVEP-SP, and (F) ClinVEP-SP.

**Extended Data Fig. 3.**
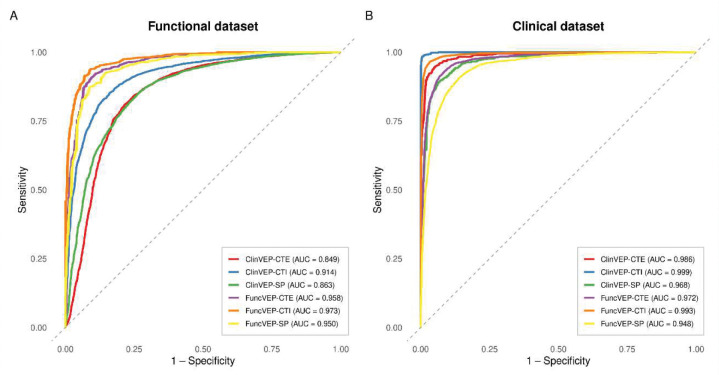
Receiver operating characteristic (ROC) curves for the six models evaluated on the functional and clinical datasets. ROC curves for all six models are shown on a single plot. (A) Performance on the functional dataset. (B) Performance on the clinical dataset.

**Fig. 3 |:**
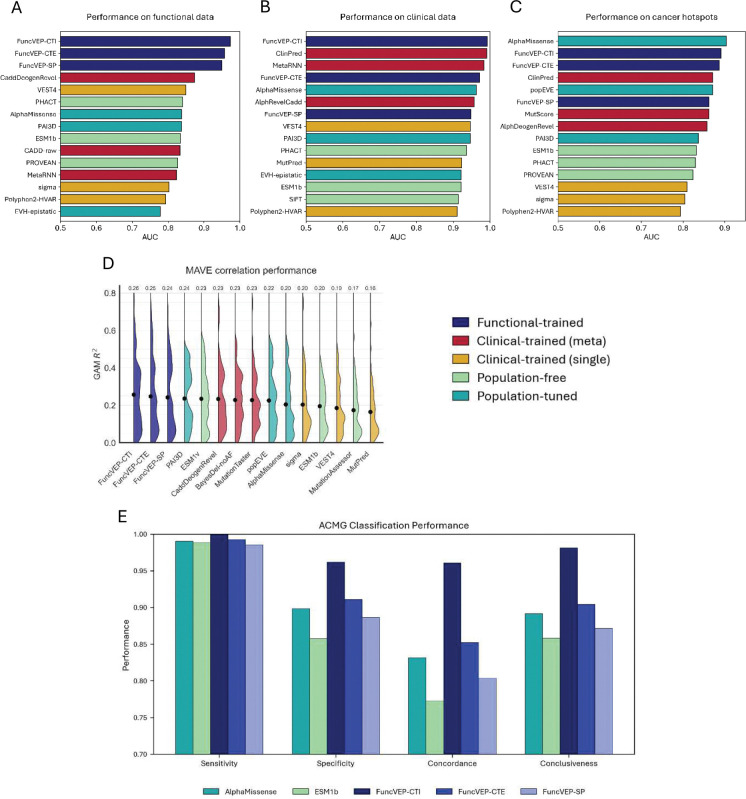
Comprehensive benchmarking of FuncVEP and existing VEPs across diverse datasets. All benchmarking evaluations are reported as AUC or GAM R^2^. Top three performers per category are shown for each benchmark. Bar plots display AUC values for classification benchmarks, with tools on the y-axis and AUC on the x-axis: (A) Performance on the functional dataset, composed of functionally damaging and neutral missense variants, balanced by gene and class. (B) Performance on the clinical dataset, consisting of high-confidence ClinVar missense variants (≥2-star review status), similarly balanced. (C) Performance on cancer hotspot data, distinguishing 878 somatic missense mutations from 1,756 matched rare missense controls across 209 known cancer driver genes. (D) Violin plots show correlations between model scores and MAVEs, reported as GAM R^2^ across 58 studies. Tools are shown on the x-axis and GAM R^2^ on the y-axis. The width of each violin reflects the density of studies at that correlation level. Dots represent the mean R^2^ per tool, which is also displayed numerically above each plot. (E) Bar plots summarize ACMG classification performance. The x-axis lists evaluation metrics (sensitivity, specificity, concordance, and conclusiveness, defined as 1 – VUS-mid ratio); the y-axis shows metric values. Within each metric, bars for five tools (AlphaMissense, ESM1b, and three FuncVEP models) are color-coded by tool.

We benchmarked all three FuncVEP and ClinVEP models alongside 47 VEPs (45 plus two additional PrimateAI models) using independent evaluations on functional and clinical datasets (Figure 3; Extended Data Figures 2, 3). The functional dataset is comprised of missense variants labeled as functionally damaging or neutral based on multiplexed assays, plus text-mined and curated experimental evidence, while the clinical dataset consisted of high-confidence pathogenic and benign missense variants from ClinVar (≥2-star review status). Both datasets were balanced per gene and class, with only 6.7% variant overlap between them. Importantly, any variants present in the training sets of FuncVEP or ClinVEP models were excluded from their respective test sets, ensuring that this limited overlap did not influence performance estimates (Supplementary Information: VEP score omission and circularity elimination).

**Extended Data Fig. 4.**
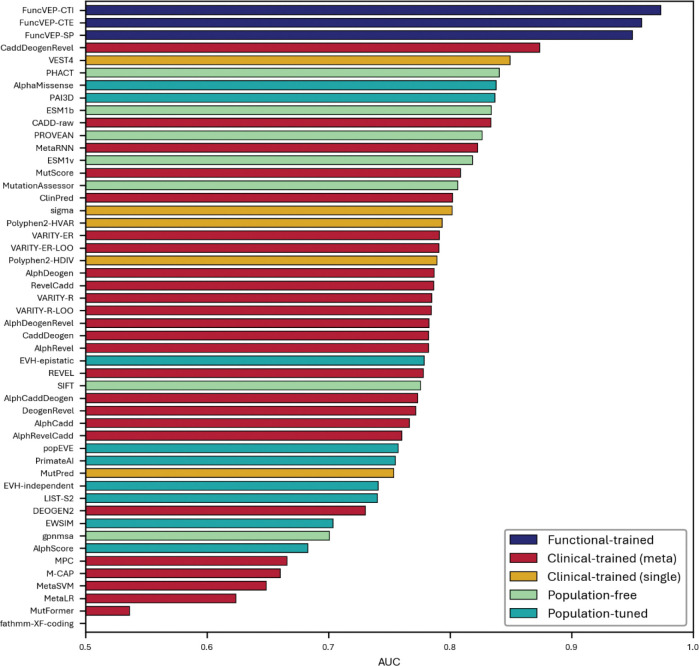
Performance of 50 VEPs on the functional dataset. Bar plot showing the AUC of each variant effect predictor on the functional dataset. The x-axis represents AUC values, and the y-axis lists the individual tools.

On the functional dataset, all three FuncVEP models outperformed existing tools by a substantial margin, achieving 7.4% to 13.5% higher accuracy. FuncVEP-CTI achieved the highest performance with an area under the receiver operating curve (AUC) of 0.97, followed by FuncVEP-CTE (AUC = 0.96) and FuncVEP-SP (AUC = 0.95). For comparison, the next best-performing tool, CaddDeogenRevel—an ensemble score combining CADD, DEOGEN2, and REVEL—reached an AUC of 0.87 (Figure 3A; Extended Data Fig. 4 & Supplementary Table 4).

**Extended Data Fig. 5.**
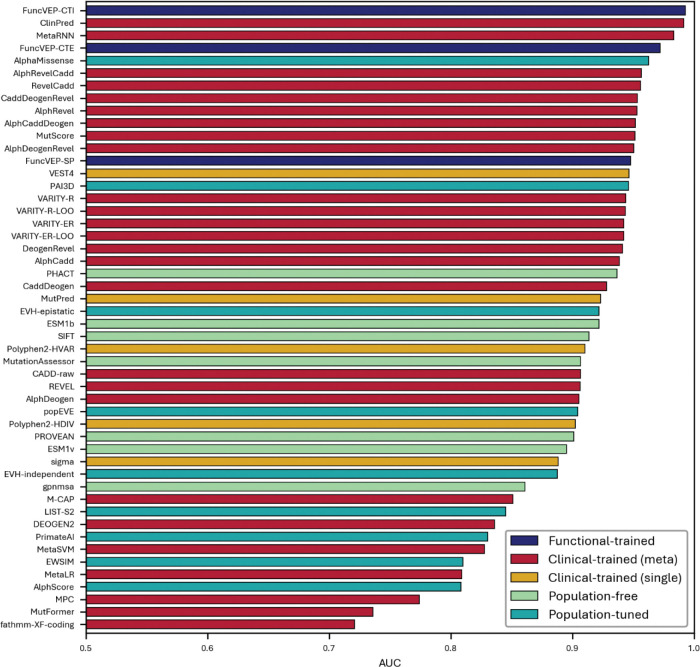
Performance of 50 VEPs on the clinical dataset. Bar plot showing the AUC of each variant effect predictor on the clinical dataset. Bars are color-coded by tool category. The x-axis represents AUC values, and the y-axis lists individual predictors.

Likewise, on the clinical dataset, FuncVEP-CTI (AUC = 0.9930) outperformed all 47 VEPs, including the top-performing clinical-trained tools such as ClinPred (AUC = 0.9917) and MetaRNN (AUC = 0.9833), despite having no exposure to clinical data during training. FuncVEP-CTE (AUC = 0.9720) and FuncVEP-SP (AUC = 0.9476) also ranked among the highest-performing tools (Figure 3B; Extended Data Fig. 5 & Supplementary Table 5).

### FuncVEP achieves state-of-the-art correlation with multiplex assays of variant effect

**Extended Data Fig. 6.**
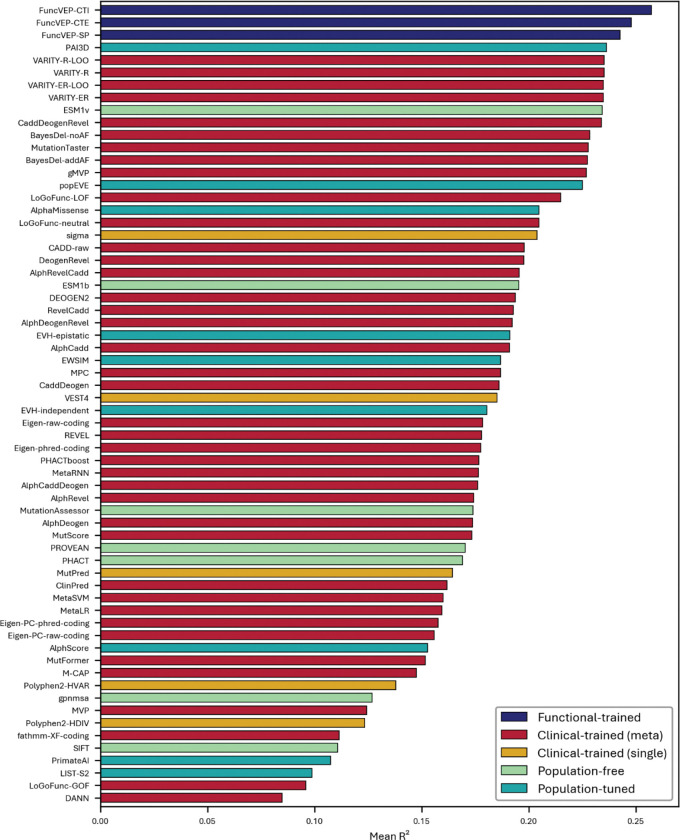
Correlation (GAM R^2^) of 50 VEPs with calibrated MAVE datasets. Bar plot showing the average R^2^ value of each variant effect predictor across MAVE studies, calculated using a generalized additive model (GAM).

MAVEs measure the functional impact of thousands of variants across a gene, providing a scalable and annotation-independent benchmark with broad sequence coverage (see Supplementary Information: VEP score omission and circularity elimination). We compiled scores from 58 MAVE studies from MaveDB spanning 58 unique genes and assessed agreement between predictor outputs and experimental measurements by calculating the coefficient of determination (R^2^) for each study, then averaging across studies to obtain an overall correlation^15^. Under the generalized additive model (GAM), FuncVEP-CTE achieved the highest mean correlation (0.257), followed by FuncVEP-CTI (0.248) and FuncVEP-SP (0.243), outperforming all other tools tested (Figure 3D; Extended Data Fig. 6 & Supplementary Tables 6 and 7). Among existing predictors, the top performing tool was PrimateAI-3D (0.236). VARITY models were excluded from the benchmark due to their use of MAVE scores as input features.

### FuncVEP discriminates disease-causing *de novo* mutations

Trio-sequencing revealed that *de novo* missense mutations (DNMs) are a major contributor to severe pediatric disorders^16^. To evaluate the power of discrimination of variant effect predictors against pathogenic versus benign DNMs, we curated datasets for developmental disorders (DD) and neurodevelopmental disorders (NDD) (see Supplementary Information: VEP score omission and circularity elimination). The DD dataset includes 3,400 disease-causing and 113 benign DNMs^17,18^, while the NDD dataset contains 2,536 pathogenic and 348 benign DNMs^19,20^ (Supplementary Table 8; Supplementary Information: *De novo* datasets).

**Extended Data Fig. 7.**
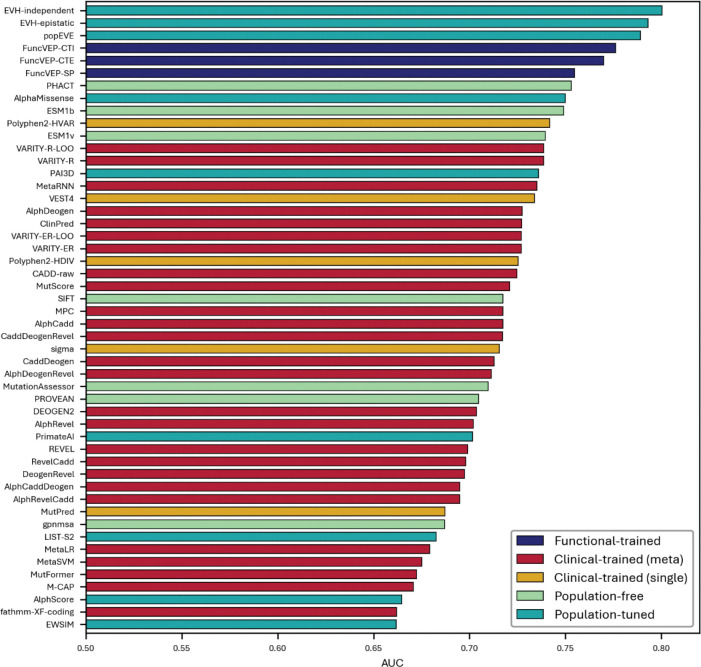
Performance of 50 VEPs on *de novo* missense variants from developmental disorder (DD) cohorts. Bar plot showing the AUC of each variant effect predictor on the developmental disorders *de novo* dataset. The x-axis represents AUC values, and the y-axis lists individual predictors.

**Extended Data Fig. 8.**
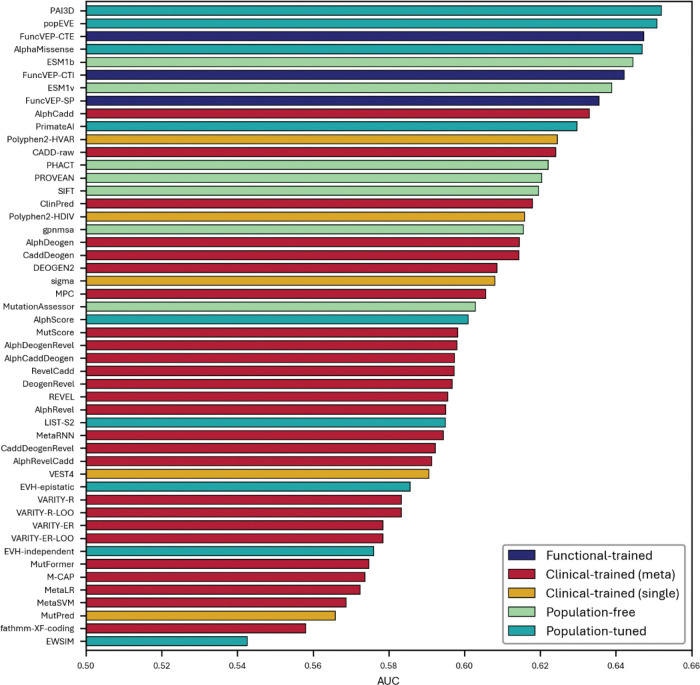
Performance of 50 VEPs on de novo missense variants from neurodevelopmental disorder (NDD) cohorts. Bar plot showing the AUC of each variant effect predictor on the neurodevelopmental disorders *de novo* dataset. The x-axis represents AUC values, and the y-axis lists individual predictors.

**Extended Data Fig. 9.**
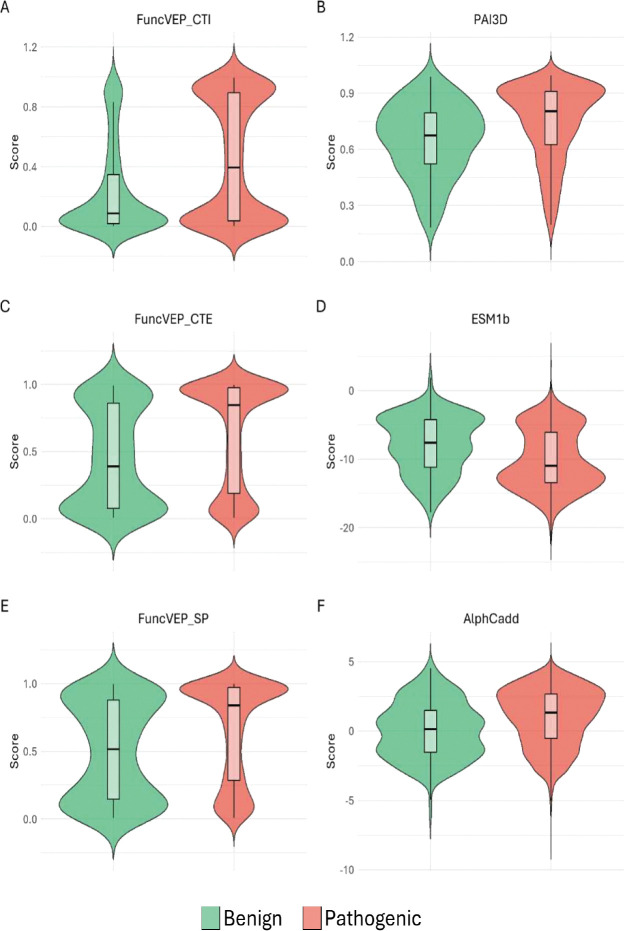
Distribution of model scores on the neurodevelopmental disorders *de novo* dataset. Violin plots display the distribution of pathogenic (red) and benign (green) variants from the NDD *de novo* variants across the scores of the best performing predictor from each category and all FuncVEP models. For each class, the violin’s width is proportional to density, while the overlaid white boxplot indicates the median and inter-quartile range. The x-axis shows the variant class and the y-axis shows the raw model score. Panels correspond to individual models: (A) FuncVEP-CTI, (B) PAI3D, (C) FuncVEP-CTE, (D) ESM-1b, (E) FuncVEP-SP, and (F) AlphCadd.

In the DD dataset, the highest-performing tools were popEVE (AUC = 0.79) and FuncVEP-CTI (AUC = 0.78), followed closely by FuncVEP-CTE (AUC = 0.77) (Extended Data Fig. 7 & Supplementary Table 9). In the NDD dataset, AUC values were generally lower, with FuncVEP-CTE, popEVE, PrimateAI-3D and AlphaMissense all performing similarly (AUCs ≈ 0.65) (Extended Data Fig. 8 & Supplementary Table 10). Although both datasets are inherently noisy due to the assumption that all case variants are pathogenic and all control variants are benign (Extended Data Fig. 9), all FuncVEP models maintained consistent top-tier performance across both datasets. Overall discrimination was modest for all tools, reflecting the challenging nature of the data.

### FuncVEP distinguishes somatic driver mutations in cancer hotspots

**Extended Data Fig. 10.**
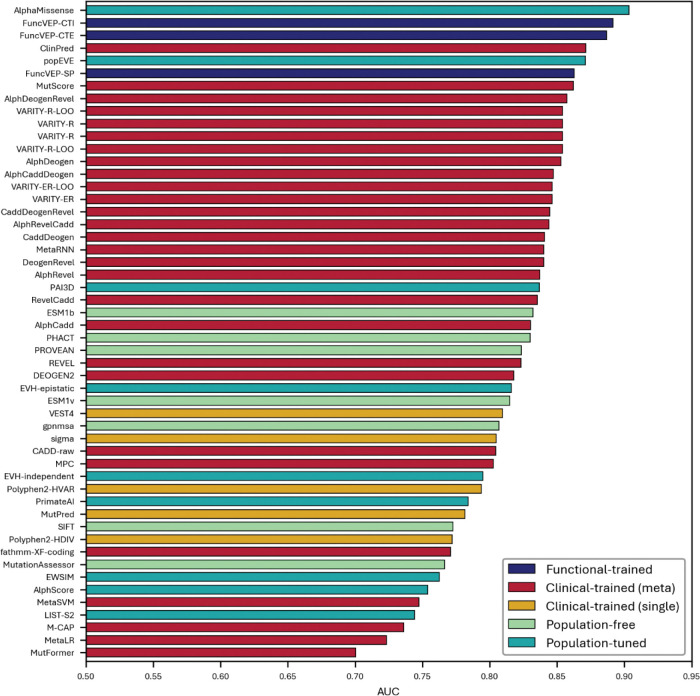
Performance of 50 VEPs on somatic missense variants in cancer hotspots. Bar plot showing the AUC of each variant effect predictor on the cancer hotspot dataset. The x-axis represents AUC values, and the y-axis lists individual predictors.

To assess performance on somatic driver mutations, we benchmarked all VEPs using a dataset of 878 cancer hotspot variants, with 1,756 matched rare missense variants from the DiscovEHR population cohort as controls^21^ (Supplementary Table 11; Supplementary Information: VEP score omission and circularity elimination). Among all tools tested, AlphaMissense demonstrated the highest performance (AUC = 0.90), followed closely by FuncVEP-CTI and FuncVEP-CTE (both AUC = 0.89) (Figure 3C; Extended Data Fig. 10). FuncVEP-SP also performed strongly (AUC = 0.86), outperforming most existing predictors. For comparison, the top clinical-trained meta-predictor was ClinPred (AUC = 0.87), the top population-free model was ESM1b (AUC = 0.83), and the top clinical-trained single predictor was VEST4 (AUC = 0.81) (Supplementary Table 12).

### Calibrated FuncVEP predictions improve ACMG classification performance by reducing uncertainty

Uncertainty is a major challenge in clinical genetics, with inconclusive reports reaching 76.2% in large gene panels and 22.5% in WES/WGS, largely due to VUS^22^. Reducing VUS, particularly VUS-mid, can improve diagnostics by increasing the rate of conclusive reports. To enable FuncVEP outputs to be used within standardized classification frameworks and to assess their impact on uncertainty, we calibrated prediction scores to ACMG/AMP evidence strengths under PP3/BP4 criteria^10,23,24^ (Supplementary Information: ACMG classification performance; VEP score omission and circularity elimination). We then compared the calibrated outputs of the three FuncVEP models with those of two leading non-clinical-trained tools, AlphaMissense and ESM1b. After annotating clinical dataset variants with PM1 and BS1, in addition to the PP3/BP4 criteria, AlphaMissense and ESM1b showed lower overall concordance with clinical annotations (0.83 and 0.77, respectively) and a notably higher proportion of uncertain classifications (VUS-mid rates of 11% and 14%, respectively). In contrast, FuncVEP-CTI achieved the highest concordance (0.96) while substantially reducing uncertainty (VUS-mid rate of 2%, which translates to 98% conclusiveness) (Figure 3E; Supplementary Table 13–15). FuncVEP-CTE and FuncVEP-SP also performed strongly, with concordances of 0.85 and 0.80 and VUS-mid rates of 10% and 13%, respectively. These results demonstrate that FuncVEP achieves high concordance under calibrated ACMG thresholds while markedly reducing VUS-mid, highlighting its potential to improve diagnostic yield by minimizing uncertainty.

To further assess classification conclusiveness, we reanalyzed a previously published gnomAD dataset of variants in disease-associated genes^5^, originally assessed using the clinical-trained tool REVEL. FuncVEP-CTI reduced the VUS-mid rate from 13.8% to 6.1%, underscoring its reliability and value for diagnostic DNA sequencing (Supplementary Table 16).

### FuncVEP uncovers the phenotype spectrum of IEI genes in the UK Biobank

We investigated the phenotypic spectrum of 509 IEI-associated genes by performing a PheWAS on 1597 phenotypes using an additive genetic model in the UK Biobank^25,26^ (Supplementary Table 17). Variants were analyzed using 13 genetic masks grouped into four categories: (1) ACMG-classified, (2) predicted damaging missense, (3) high-impact, and (4) synonymous (see Methods). For ACMG classification, we used AAVC^27^, after the incorporation of FuncVEP predictions under PP3/BP4 criteria. 490/509 genes harbored at least one variant under any mask.

A total of 200 gene–phenotype associations surpassed the Bonferroni-adjusted threshold (p < 1.02 × 10^−4^; 0.05/490) (Supplementary Information: Threshold for reporting). Of these, 85 represented replications of the primary disease phenotype (e.g., *BRCA1*–female breast cancer), 54 reflected associations with known complications of the primary condition (e.g., *MSH6*–intestinal/peritoneal adhesions, potentially associated with colorectal cancer surgery), 11 indicated dominant presentations of phenotypes typically considered recessive (e.g., *C1QC*–C1q deficiency 3 in autosomal recessive form, and increased risk for *S. aureus* infection in dominant form), and 50 were novel gene–phenotype associations, of which two achieved genome-wide significance (p < 1.02 × 10^−6^), *SAMHD1* for mature B-cell malignancies and *DCLRE1B* for polyneuropathies (Table 1 and Supplementary Table 18). For the dominant presentation of recessive diseases, no individuals were identified as compound heterozygotes for the associated variant mask. In the case of *C1QC* and S. aureus infection, 1 out of 11 affected individuals was homozygous, while the remaining 10 were heterozygous. Among the 50 novel associations, 24 were identified through ACMG-classified variants, 14 through high-impact, and 12 through missense variants predicted as damaging by FuncVEP-CTE.

The 50 novel associations involve 47 genes and span 13 phenotype categories: neoplastic (n=12), cardiovascular (10), endocrine/metabolic (5), musculoskeletal (5), neurologic (4), sense organs (3), genitourinary (2), infections (2), symptoms (2), congenital (1), dermatologic (1), gastrointestinal (1), and pregnancy-related (1). Of these 47 genes, 41 were not previously known to be associated with any phenotype other than IEI.

We then examined the 50 novel associations in the MSM African (AFR, *n* = 19,856), Admixed American (AMR, *n* = 8,531), and European (EUR, *n* = 21,680) cohorts (https://icahn.mssm.edu/research/ipm/programs/mount-sinai-million). Eight displayed nominal significance (p < 0.05): one in AMR (*RFXANK* with macular degeneration), two in EUR (*SEMA3E* with heart block and *STAT6* with hypothyroidism), and five in AFR (*NOD2* with hepatitis, *CLPB* with lower gastrointestinal tract malignancies, *SOCS1* with osteoporosis, and *MAN2B2* with bronchus and lung cancer) (Supplementary Table 19). The limited replication in MSM likely reflects its smaller sample size and greater genetic diversity.

Importantly, seven of the novel gene–phenotype associations identified in this study are supported by prior evidence from family-based linkage analyses or biobank-scale genome-wide association studies (GWAS). For instance, linkage analysis in two multigenerational families mapped autosomal dominant early-onset juvenile open-angle glaucoma (MIM: GLC1J, 608695) to chromosome 9q22.3, a region that includes *TGFBR1*^28^. In our analysis, *TGFBR1* is associated with glaucoma, potentially establishing a causal link with GLC1J, a connection not previously reported despite the gene’s presence within the critical region.

Likewise, the causal gene for autosomal dominant Parkinson’s disease (PD; PARK11; MIM: 607688) was initially proposed to be *GIGYF2* based on five missense variants identified in affected families. However, subsequent studies have challenged its role in PARK11^29,30^. Notably, all five variants are predicted to be neutral by all FuncVEP models. Interestingly, *ALPI*, which was associated with PD in our analysis, lies within 0.2 megabases of *GIGYF2*, raising the possibility that PARK11 may instead result from high-impact *ALPI* mutations.

Furthermore, five novel associations overlap with previously reported GWAS signals for the same phenotypes: *TERT* (abdominal aortic aneurysm)^31^, *LPIN2* (coronary atherosclerosis)^32^, *MCM10* (hypercholesterolemia)^33^, *NFATC1* (hypothyroidism)^34^, and *ERBIN* (type 2 diabetes)^35^, further supporting the involvement of these genes with the respective phenotypes.

To further characterize the clinical relevance of these associations, we estimated penetrance for each gene–phenotype pair. Quantifying disease risk from genetic variation is critical to the goals of precision medicine. Many complex conditions, such as cardiovascular diseases, type 2 diabetes, neurological disorders, and various cancers, are influenced by both genetic and environmental factors and typically do not follow simple Mendelian inheritance^36^. To account for this variability, we applied Bayesian estimation at the median age of 58 to quantify the probability of phenotype expression for 50 novel gene-phenotype associations. The highest penetrance estimate was observed for *MCM10* and hypercholesterolemia (88.9%), followed by *ERBIN* and type 2 diabetes (64.8%), and *SNX10* and female breast cancer (53.1%). In contrast, the lowest penetrance was seen for *ALPI* and optic atrophy (0.8%) (Table 1).

### FuncVEP reveals the genetic epidemiology of inborn errors of immunity

We assessed the inherited burden of 509 IEI genes across eight genetic ancestries by conducting a genetic epidemiology study leveraging allele frequency data from gnomAD^2,26^ (Supplementary Table 20). We used AAVC^27^ to classify variants, incorporating FuncVEP predictions under the PP3/BP4 criteria, and calculated carrier frequency (CrF) and genetic prevalence (GP) for pathogenic (P), likely pathogenic (LP), and VUS-high (VUS-H) variants.

The top three genes with the highest average CrF and GP for P+LP variants across eight genetic ancestry groups were: for CrF, *CFTR* (AR, cystic fibrosis, 1 in 16; 456 million individuals), *G6PD* (XLR, G6PD deficiency class I–favism, 1 in 20; 360 million), and *DIAPH1* (AR, DIAPH1 deficiency, 1 in 32; 230 million); and for GP, *NOD2* (AD, Blau syndrome, 1 in 47; 155 million), *G6PD* (XLR, G6PD deficiency class I–favism, 1 in 55; 135 million), and *CFHR5* (AD/AR, factor H–related protein deficiency, 1 in 60; 123 million) (Supplementary Table 20).

**Fig. 4 |:**
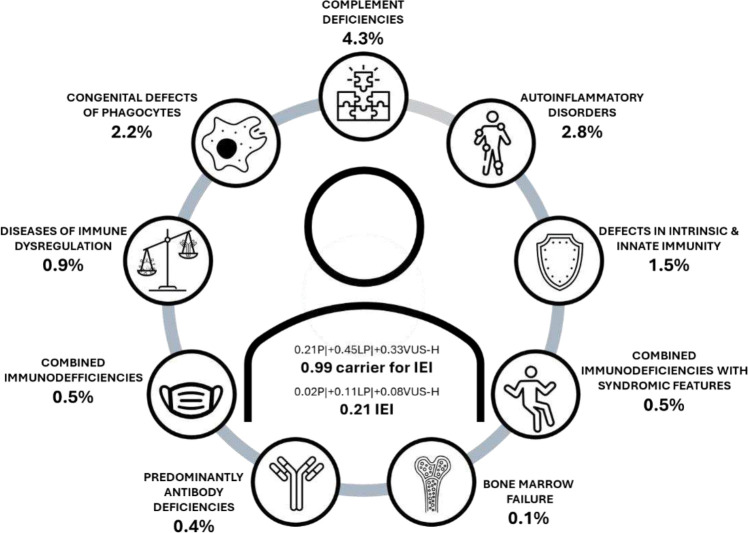
Genetic epidemiology of IEI genes. P+LP+VUS-H variants per individual for 509 IEI genes, and GP of P+LP variants according to nine IEI-based disease groups on average for the eight genetic ancestries.

We next calculated the mean probability of an individual carrying any P, LP, or VUS-H variant based on CrF across all genetic ancestries: 0.21 for P, 0.66 for P+LP, and 0.99 for P+LP+VUS-H. At the genotype level (cumulative GP), the average individual harbored 0.02 P, 0.12 P+LP, and 0.21 P+LP+VUS-H genotypes, consistent with heterozygous dominant, homozygous recessive, or hemizygous X-linked inheritance. The probability of not carrying any P+LP variant was 52% (3.8 billion individuals), and of not carrying any P+LP+VUS-H variant was 37% (2.7 billion). (Fig. 4, Supplementary Table 21).

We further assessed the genetic prevalence ranking of the newly identified gene–phenotype associations relative to established Mendelian disease genes for the corresponding phenotype. For example, *SNX10* is identified as a novel breast cancer susceptibility gene, among 13 known genes, and its high-impact variants are potentially carried by 6.1 million individuals worldwide, making it the sixth most common gene associated with breast cancer risk. We present the GP ranking of all 47 genes within their respective disease categories (Table 1 and Supplementary Table 22). Notably, several associations rank first in their phenotype groups: *ALPI* in optic atrophy (1/41), *CLPB* in colorectal cancer (1/18), *CTC1* in prostate cancer (1/10), *FANCM* in thoracic aneurysm (1/36), *FNIP1* in atrial fibrillation and flutter (1/4), *KMT2D* in non-melanoma skin cancers (1/4), *RIPK3* in generalized osteoarthritis (1/4), *SAMHD1* in malignant mesothelioma (1/2), and *SLC29A3* in atrial septal defect (1/7).

## Discussion

Computational interpretation of human genetic diversity is central to precision medicine, with missense variants comprising the largest class of coding variation^4^. To improve missense classification, we developed FuncVEP-CTI, FuncVEP-CTE, and FuncVEP-SP, the first family of variant effect predictors trained on balanced, diverse, and biologically grounded functional data. Comprehensive benchmarking demonstrates that FuncVEP models achieve state-of-the-art performance, surpassing 47 established predictors across functional, clinical, MAVE, *de novo*, and somatic driver mutation datasets. (Supplementary Tables 4 and 5)

To determine if functional training, rather than feature design, drives the improved performance, we trained parallel ClinVEP models on clinical data using the same feature set. Although all three ClinVEP models perform exceptionally on the clinical dataset, with ClinVEP-CTI achieving a near perfect AUC of 1.00, they do not generalize as well to functional data, where performance drops to an AUC of 0.91. This parallels the limitations observed in other clinical-trained tools and likely reflects inflated performance estimates when models are evaluated on clinical datasets (Supplementary Tables 23). The discrepancy is likely due to overfitting, as suggested by unstable thresholds across datasets and limited generalizability, and may stem from the disproportionate reliance on constraint and conservation metrics observed in clinical-trained models (Supplementary Tables 4 & 5). In contrast, functional data enable FuncVEP to learn a broader spectrum of variant impact patterns by capturing mechanistic consequences across the genome.

FuncVEP has certain limitations. Because it is trained to classify variants as either damaging or neutral, without modeling specific mechanisms such as LOF or GOF, it does not provide information about the directionality of a variant’s effect. Additionally, as a classifier trained on binary labels, FuncVEP is not designed to estimate the magnitude of functional impact, which would require a regression-based approach.

Depending on the application, we recommend FuncVEP-CTI for clinical use, where minimizing uncertainty is critical for diagnostic decision-making. FuncVEP-CTE is better suited for research applications such as burden testing and PheWAS, given its independence from clinical-trained tools. FuncVEP-SP provides unique value in scenarios requiring predictions independent of allele frequency, both directly and indirectly, such as evaluating common yet potentially damaging variants.

We showed that FuncVEP enables biobank-scale analyses, including phenome-wide association studies, by facilitating the discovery of novel genotype–phenotype relationships. Integrating FuncVEP-CTE predictions into the analysis of 490 IEI genes in the UK Biobank, we identified 50 previously unreported gene–phenotype associations. Among these, eight were replicated in the MSM cohort across three genetic ancestries, two reached genome-wide significance, two overlapped with prior linkage loci, and five coincided with known GWAS signals, resulting in 17 associations supported by independent lines of evidence. The remaining 33 associations are considered suggestive (p < 1.02 × 10^−4^) and warrant further investigation. Notably, 12 of the 50 were discovered exclusively through predicted damaging missense variants, while 24 were captured only when using ACMG-classified variants that incorporated FuncVEP-CTE annotations (Table 1, Supplementary Table 14).

The distinction between biallelic and monoallelic inheritance is a key driver of phenotypic diversity in inherited disease. Consistent with semi-dominance, our results suggest that biallelic mutations in IEI genes predominantly yield immune phenotypes, whereas monoallelic variants in the same genes are associated with a broader spectrum of adult-onset disorders. This allelic architecture helps reconcile the gene pleiotropy observed across our PheWAS together with genetic epidemiology, provides a foundation for advancing public health genomics in inherited disorders.

Our findings demonstrate that training on functional evidence enables state-of-the-art performance while reducing circularity, establishing a promising paradigm for missense interpretation. Functionally grounded approaches hold potential not only for improving variant classification in rare disease diagnostics but also for uncovering genotype–phenotype relationships beyond IEI genes. We anticipate that functional training approaches will become increasingly central and continue to improve missense classification.

## Methods

### Functional Dataset

To construct a high-confidence and biologically diverse functional dataset, we aggregated missense variants from three primary sources: text-mined functional evidence from ClinVar using a fine-tuned BioBERT model, manually curated data from the literature, and high-throughput mutagenesis studies from MaveDB^15,37^. The final balanced dataset was constructed through a multi-step integration and filtering process.

For text-mined evidence, 250,819 ClinVar submissions were screened for 45 keywords related to functional evidence (Supplementary Table 24). A BioBERT biomedical language model was trained on over 6,000 manually labeled entries and achieved 97% classification accuracy. This model identified 19,922 functionally described variants, of which 10,017 were missense (Supplementary Information: Text-mined functional annotations).

For literature curation, 5,804 PubMed articles published between 2018 and 2023 were queried using “missense” and “function,” and 42 large-scale studies across 30 genes were selected. These studies contributed functional annotations for 28,323 unique missense variants (Supplementary Table 25; Supplementary Information: Manually curated functional data from literature).

For MAVEs, we collected variant effect scores from 274 studies from MaveDB and calibrated them with ClinVar ≥2-star variants for clinical relevance. We selected 26 studies across 11 genes that passed the Bonferroni-corrected performance threshold, resulting in 36,006 unique variants (Supplementary Table 26; Supplementary Information: Multiplex assays of variant effect).

To improve class balance, we also included a set of proxy benign variants from gnomAD v4.1, used exclusively for training^2^. Singletons (allele count = 1) and variants overlapping with clinical or functional datasets were excluded. Each retained variant was labeled as benign and assigned a sample weight based on log-transformed and min-max normalized allele frequency to reflect confidence in benignity (Supplementary Information: Proxy benign variants).

A reliable, balanced, and biologically diverse dataset was constructed from these sources through a multi-step integration process (Supplementary Information: Variant selection and functional dataset construction), resulting in 18,556 missense variants (9,357 damaging, 9,199 neutral), of which 6,641 benign labels came from proxy data, spanning 1,576 genes.

### Clinical Dataset

Variants reported in ClinVar as of January 20, 2025, aligned to the GRCh38 (hg38) reference genome, were downloaded from the ClinVar database (https://www.ncbi.nlm.nih.gov/clinvar/)^6^. Variants with a review status of zero stars (no assertion criteria provided) or one star (criteria provided, single submitter) were excluded. Only variants with a review status of two stars (criteria provided, multiple submitters, no conflicts), three stars (reviewed by expert panel), or four stars (practice guideline) were retained to ensure higher confidence in clinical significance annotations (Supplementary Information: Clinical dataset).

We retained only missense variants, excluding variants with ambiguous, conflicting, or VUS annotations. Remaining variants, labeled as pathogenic, likely pathogenic, benign, or likely benign, were grouped into binary classes, with final labels simplified as pathogenic and benign. To ensure the selected variants reflect missense effects as the primary molecular mechanism, we excluded those predicted to disrupt splicing (SpliceAI score > 0.2)^38^. After gene and label balancing, the final clinical dataset consisted of 11,718 missense variants, including 5,859 labeled as benign and 5,859 labeled as pathogenic. This dataset was used to train the ClinVEP models and evaluate the FuncVEP models.

### Features

We compiled a total of 571 features spanning diverse biological levels and data sources to maximize predictive power and interpretability. These features fall into five types: VEP outputs and conservation scores, as well as residue-level, mutation-level, and gene-level features (Supplementary Information: Features).

Forty-five features were outputs of existing VEPs. Most were obtained from dbNSFP v5.0, while others were collected from their original publications or associated repositories^39^.

Twenty-four residue-level features capture the structural or constraint-related context of the mutated amino acid. These include seven constraint scores (e.g., MTR), and seventeen structural features such as relative solvent accessibility (RSA), contact number, and secondary structure class.

Twenty-four mutation-level features characterize the biochemical and evolutionary properties of the amino acid substitutions. This group includes twenty substitution matrix scores (e.g., Grantham, BLOSUM, VTML), as well as four protein language model–derived metrics computed using the ESM-2 architecture^40^ (Supplementary Information: Novel features). The latter quantifies the impact of the substitution on sequence plausibility and representation, based on differences in log-likelihood, perplexity, and embedding shift between wild-type and mutant sequences.

Gene-level features accounted for the largest group, with 465 features. These include 68 gene expression features derived from GTEx v10^41^, seven gene constraint scores of pLI, missense, loss-of-function and synonymous Z-scores phi, RVIS, *S*_het_, and 389 features from large-scale gene knock-out studies in diverse cell types. Folding stability was included as an additional structural metric at the gene level.

Finally, we included 13 conservation scores that quantify evolutionary constraint at the nucleotide or amino acid level, such as GERP++, PhyloP, and PhastCons.

To maintain independence from other ACMG criteria and avoid circularity, we excluded features that could overlap with classification rules, such as allele frequency, splicing impact scores, clinically relevant domain annotations, and MAVE scores.

### Model Development

We trained six binary classification models to predict the pathogenicity of missense variants using the LightGBM framework^13^. Three models (FuncVEP-CTI, -CTE, -SP) were trained on the functional dataset, and three mirror models (ClinVEP-CTI, -CTE, -SP) were trained on the clinical dataset. Each model varied in the inclusion of variant effect predictor features: CTI models included all available tools, CTE models excluded clinical-trained predictors, and SP models excluded all predictors entirely. Feature dimensions vary depending on predictor inclusion: CTI models use 558 features, CTE models 527, and SP models 513 (Supplementary table 2). All models were trained using a 90/10 train-test split with Optuna hyperparameter optimization set to 50 trials, and the final configuration was selected based on AUC performance on the held-out evaluation set ^42^.

To prevent circularity, we implemented a multi-level score omission strategy. For any variant included in the training set of a given tool, we removed its score from the corresponding feature. This exclusion was applied recursively to account for meta-predictors and their component tools (Supplementary Information: VEP score omission and circularity elimination).

To address informative missingness, we performed machine learning–based imputation for 53 features exhibiting biased missingness. LightGBM regressors were trained on 420,731 unlabeled missense variants to estimate missing scores, using the remaining features as input. This approach preserved natural score distributions and avoided artifacts caused by mean or median imputation methods (Supplementary Information: Feature imputation).

Sample weights were assigned to downweight proxy benign variants, which were weakly labeled as benign and used only in functional model training. All other variants from functional and clinical sources were assigned a weight of 1 (Supplementary Information: Sample weight assignment).

### Benchmarking

We evaluated 47 prediction tools plus all FuncVEP and ClinVEP models, totaling 53, across five benchmarking frameworks designed to test performance on both clinical and research utility. Benchmarks were conducted on the functional and clinical datasets, MAVE scores, *de novo* mutations from developmental and neurodevelopmental disorder cohorts & population controls^2,17–20^, and somatic cancer hotspot mutations^21^.

To avoid evaluation bias, we implemented strict circularity controls. For each tool, scores were omitted for any variants included in its own training set or the training sets of any component tools. Fourteen tools with unavailable training data were excluded from benchmarking. FuncVEP and ClinVEP models were evaluated on 10% held-out splits of their respective training datasets when benchmarked on the corresponding dataset used for training.

Performance was assessed using AUC and accuracy for classification tasks using pROC package, and a generalized additive model for MAVE score correlation performed in RStudio^43,44^. For the MAVE benchmark, tools were evaluated based on their correlation with each calibrated MAVE study individually and by their mean R^2^ across studies. Correlations were only computed for tool–study pairs with at least 10 available scores; pairs with fewer than 10 variants were excluded. For *de novo* and cancer benchmarks, AUCs were computed to assess discriminatory power between disease and control variants.

### Calibration of model scores for ACMG PP3/BP4 criteria

To enable the use of model outputs as computational evidence under ACMG/AMP guidelines, we calibrated continuous prediction scores to PP3/BP4 evidence strengths. Calibration followed the probabilistic framework, in which local positive predictive value (PPV) and negative predictive value (NPV) are estimated across score intervals^23^. These values were then mapped to supporting, moderate, or strong evidence levels using the likelihood ratio thresholds, assuming a prior pathogenicity probability of 10% and a positive likelihood ratio threshold of 350^24^.

Calibration was performed using high-confidence ClinVar missense variants (≥2-star review status; January 2025 release) combined with representative population variants from gnomAD v4.1^2,6^. The same calibration procedure was applied to AlphaMissense and ESM1b to enable direct comparison.

ACMG classification performance was then measured on the clinical dataset, excluding variants annotated with the BA1 criterion by AAVC or lacking scores from any of the tools, resulting in 3,394 pathogenic and 3,605 benign variants.

### Phenome-wide association study

We analyzed data from the UK Biobank, which includes approximately 500,000 individuals aged 40–69 years at recruitment^25^. We excluded participants who were outliers for genotype missingness or heterozygosity (UKB datafield: p22027), exhibited discordance between self-reported sex (p31) and chromosomal sex (p22001), or showed evidence of sex chromosome aneuploidy (p22019). Only individuals meeting the overall quality control criteria (p32064) were retained, yielding a final cohort of 413,892 participants. We retained variants for which at least 90% of genotypes had a sequencing depth (DP) greater than 10 (excluding OQFE variants). Variants were removed if they had >10% missingness or showed significant deviation from Hardy–Weinberg equilibrium (p < 1 × 10^−15^). For phenotype definitions, we used the PheCodeX repository, which provides a curated mapping of ICD-10 codes^45^. For case-control definitions, individuals diagnosed with a given neoplasm were assigned to the case group, while those with any other malignant neoplasm were excluded from the control group. For example, when testing breast cancer, individuals with colon or any other cancer were excluded from controls. No such exception is defined for non-neoplastic phenotypes. Furthermore, phenotypes assigned to fewer than 50 individuals in the UK Biobank were excluded from analysis. resulting in 1597 phenotypes (Supplementary Table 27).

We used an automated ACMG classifier, AAVC^27^, for the classification of 975,940 UKB variants from 509 known IEI genes. Variant sets were defined using three masks: For the analysis, we used 13 masks for variant sets: (1) P, (2) P+LP, (3) P+LP+VUS-H, (4) P+LP+VUS-H+FuncVEP-D, (5) FuncVEP-D, (6) PVS1-VS, (7) PVS1-S, (8) PVS1-M, (9) PVS1-P, (10) PVS1-VS+S, (11) PVS1-VS+S+M, (12) PVS1-VS+S+M+P and finally (13) non-splice altering synonymous variants (SpliceAI < 0.2). Here, FuncVEP-D refers to missense variants classified as P, LP, VUS-H, VUS-M, or VUS-L with FuncVEP-CTE scores exceeding 0.631. In addition, we applied four allele frequency thresholds to each mask: ≤ 0.5, ≤ 0.1, ≤ 0.01, and singletons (allele count = 1).

We performed PheWAS using Regenie v4.1^46^ with Firth logistic regression and standard error estimation (firth and firth-se options), the approximate Firth method (approx option), a p-value threshold of 0.01, and the maximum mask-building strategy (build-mask max option), adjusting for sex, age, smoking status, and the first 10 principal components as covariates. Of the 509 known IEI genes, 490 contained at least one damaging variant under one of the defined masks. Genes were excluded from analysis for a given phenotype if fewer than five individuals in either the case or control group carried a damaging variant. Gene-level associations were reported at a Bonferroni-adjusted threshold of p < 1.02 × 10^−4^ (0.05/490). To evaluate test statistic inflation, we used synonymous variants filtered at an allele frequency threshold of 0.01 to compute the genomic inflation factor across phenotype groups (Supplementary Table 28).

We performed PheWAS in AFR, AMR, and EUR cohorts of MSM to validate novel gene–phenotype associations identified in the UK Biobank. Ancestry-specific exome sequencing cohorts were generated based on genetic similarity to HapMap 3 populations^47^. A high-quality dataset was obtained after excluding gender discordant and duplicate samples, or those with exome 20X coverage < 80% and contamination >5%. Genotypes with DP > 10 or genotype quality > 20 were retained and variants with a Phred-scaled quality score < 20, a missingness >10% or a deviation from HWE with a *P* < 1×10^−15^ were excluded. Using the variant masks and phenotype definitions described above, and allele frequency thresholds of 0.01, 0.001, and 0.0001, PheWAS was conducted using SAIGE v.1.4.0, adjusting for age, sex, and the first ten principal components as covariates in each ancestral group^48^.

### Genetic epidemiology study

High-coverage (Missingness < 0.5) allele frequencies were obtained from gnomAD v4.1^2^. The cumulative allele frequency (CAF) for each gene was calculated by summing the allele frequencies of variants classified as P, P + LP, P + LP + VUS-H, or from the associated variant set. CrF and genetic GP were then estimated using CAF and Hardy–Weinberg equilibrium assumptions for autosomal genes. For X-linked recessive genes, GP was calculated as (CAF*(CAF+1))/2 and for X-linked dominant genes as (1-CAF)*CAF+(CAF*(CAF+1))/2. Ancestry-specific population sizes were derived from a previous publication^5^. For the GP ranking, genes were curated from OMIM (https://omim.org/) based on their established causality for the newly associated phenotypes. GP was calculated using P+LP variants for both the curated genes and the corresponding variant masks of the newly identified gene–phenotype associations, averaged across eight genetic ancestry groups (Supplementary Table 22).

### Penetrance estimation

Penetrance is defined as the alleles probability of expressing its associated phenotype and estimated from case carrier frequency, population carrier frequency and prevalence of the phenotype. We calculated penetrance based on Bayes’ theorem^49^

$$P(D|C)=P(D)\frac{P(C|D)}{P(C)}$$

where, D denotes the disease, C the carrier frequency, and P the probability. Penetrance is defined as P(D∣C), representing the probability of developing the disease given the presence of the risk allele. P(D) denotes the disease prevalence, i.e., the baseline probability of disease in the general population. P(C∣D) refers to the carrier frequency among affected individuals, while P(C) denotes the carrier frequency in the general population. Given the UK Biobank’s recruitment age range and median participant age of 58 years, the penetrance estimates primarily reflect risk in middle-aged individuals and may be slightly underestimated due to the cohort’s healthy volunteer bias.

## Extended Data

## Supplementary Material

Supplementary Files

This is a list of supplementary files associated with this preprint. Click to download.

• SupplementaryInformation.docx

• SupplementaryTables.xlsx

• ExtendedDataFig.docx

## Figures and Tables

**Table 1.** Novel gene–phenotype associations for inherited disease susceptibility.

Gene	Mask	Trait	OR	P	Penetrance	GP Ranking
**Neoplastic**						
*SAMHD1*	ACMG*	Mature B-cell malignancy	3.2	6.4×10^−7^	5.1%	3/89
	ACMG	Mesothelioma (all)	7.2	6.0×10^−6^	1.7%	1/3
*FAT4*	ACMG	Malignant neoplasm of fallopian tube and uterine adnexa	18.6	3.8×10^−6^	1.6%	1/1
*PSTPIP1*	ACMG	Malignant neoplasm of lips and oral cavity	6.8	3.2×10^−5^	5.2%	1/1
*CTC1*	Missense**	Prostate cancer	2.1	3.3×10^−5^	13.2%	1/10
*CLPB*	Missense	Colorectal cancer	2.9	3.6×10^−5^	11.5%	1/18
*MAN2B2*	High-impact***	Lung cancer	12.4	4.8×10^−5^	16.1%	1/1
*NOS2*	ACMG	Mesothelioma (pleura)	8.9	4.8×10^−5^	1.6%	2/3
*NCF4*	ACMG	Ovarian cancer	7.2	6.6×10^−5^	4.9%	11/12
*SNX10*	ACMG	Breast cancer (female)	11.2	7.7×10^−5^	53.1%	6/14
*LSM11*	ACMG	Cervix cancer	7.0	7.7×10^−5^	3.1%	1/1
*KMT2D*	ACMG	Non-melanoma skin cancer	10.5	9.5×10^−5^	9.5%	3/34
**Cardiovascular**						
*TERT*	Missense	Abdominal aortic aneurysm	4.9	2.6×10^−6^	5.7%	20/89
*ZBTB24*	ACMG	Aortic insufficiency	9.6	7.9×10^−6^	10.1%	2/3
*FANCM*	High-impact	Thoracic aneurysm	14.2	1.8×10^−5^	8.2%	1/36
*RNASEH2C*	Missense	Persistent atrial fibrillation	13.6	2.2×10^−5^	8.9%	3/4
*RFWD3*	High-impact	First degree atrioventricular block	4.5	3.3×10^−5^	11.0%	10/46
*LPIN2*	High-impact	Coronary atherosclerosis	2.6	3.7×10^−5^	39.7%	4/12
*DNMT3B*	Missense	Complete atrioventricular block	6.3	4.8×10^−5^	5.2%	4/46
*SEMA3E*	Missense	Heart block	4.2	5.6×10^−5^	2.7%	2/46
*NUDCD3*	ACMG	Cardiac arrhythmia and conduction disorders	3.0	8.2×10^−5^	5.4%	2/46
*FNIP1*	ACMG	Atrial fibrillation and flutter	3.4	8.9×10^−5^	43.0%	1/4
**Endocrine/Metabolic**						
*STAT6*	High-impact	Hypothyroidism	4.6	2.5×10^−5^	46.6%	5/34
*MCM10*	ACMG	Hypercholesterolemia	3.9	2.8×10^−5^	88.9%	2/12
*NFATC1*	ACMG	Hypothyroidism	3.1	5.7×10^−5^	35.4%	4/34
*ATG4A*	High-impact	Hypothyroidism	2.7	6.2×10^−5^	37.5%	7/34
*ERBIN*	High-impact	Type-2 diabetes	5.0	6.9×10^−5^	64.8%	12/61
**Musculoskeletal**						
*ARPC1B*	Missense	Gout	19.5	1.5×10^−5^	58.8%	1/1
*RIPK3*	High-impact	Generalized osteoarthritis	3.5	4.2×10^−5^	1.3%	1/4
*JAK3*	ACMG	Sacroiliitis	10.9	5.9×10^−5^	1.1%	1/1
*SOCS1*	ACMG	Osteoporosis	6.0	6.4×10^−5^	40.9%	2/4
*TCIRG1*	High-impact	Osteoarthritis	2.7	7.1×10^−5^	87.3%	2/4
**Neurological**						
*DCLRE1B*	Missense	Polyneuropathies	30.9	5.7×10^−7^	33.3%	2/5
*ALPI*	High-impact	Primary Parkinson’s disease	8.9	×10^−5^	14.3%	2/36
*GIMAP6*	High-impact	Migraine	8.9	1.8×10^−5^	21.5%	1/4
*AP3B1*	ACMG	Encephalopathy	10.5	7.3×10^−5^	1.7%	2/125
**Sense organs**						
*TGFBR1*	ACMG	Glaucoma	8.6	8.8×10^−5^	32.6%	6/24
*ALPI*	Missense	Optic atrophy	8.7	4.4×10^−5^	0.8%	1/41
*RFXANK*	ACMG	Macular degeneration	11.4	8.7×10^−5^	38.4%	3/6
**Genitourinary**						
*FNIP1*	High-impact	Chronic kidney disease	4.4	3.5×10^−5^	41.2%	1/1
*ERCC6L2*	High-impact	Chronic prostatitis	11.5	6.3×10^−5^	8.6%	1/1
**Infections**						
*NOD2*	ACMG	Hepatitis-B	10.7	6.5×10^−5^	1.7%	1/1
*BLM*	Missense	SARS-CoV-2	8.6	9.1×10^−5^	28.6%	1/1
**Symptoms**						
*DBF4*	ACMG	Bone marrow transplant	10.8	1.3×10^−5^	0.9%	1/1
*MS4A1*	ACMG	Kidney transplant	12.9	2.6×10^−5^	3.5%	1/1
**Blood/Immune**						
*FCHO1*	ACMG	Monoclonal gammopathy	18.7	4.2×10^−6^	9.1%	1/1
**Congenital**						
*SLC29A3*	Missense	Atrial septal defect	9.1	4.3×10^−5^	3.4%	1/7
**Dermatological**						
*POLE*	High-impact	Circumscribed scleroderma	6.3	4.1×10^−5^	2.7%	3/18
**Gastrointestinal**						
*IL7R*	Missense	Irritable bowel syndrome	11.1	3.1×10^−5^	46.1%	1/1
**Pregnancy**						
*SBDS*	ACMG	Preeclampsia and eclampsia	4.4	5.6×10^−5^	0.6%	1/1

* P, P+LP, P+LP+VUS-H and P+LP+VUS-H+FuncVEP-D

** FuncVEP-D

*** PVS1-VS, PVS1-S, PVS1-M, PVS1-P, PVS1-VS+S, PVS1-VS+S+M and PVS1-VS+S+M+P

## Data Availability

Precomputed FuncVEP and ClinVEP predictions for all possible missense variants are available at https://zenodo.org/records/17036008. All other data generated or analyzed during this study are included in the Supplementary Tables, including details on the external data sources.
